# Commentary: Repertoire construction for critical cross-cultural literacy of English majors: based on the research paradigm of systemic functional linguistics

**DOI:** 10.3389/fpsyg.2024.1230324

**Published:** 2024-06-11

**Authors:** Jinhua Sun, Haohan Meng, Jie Hu

**Affiliations:** ^1^College of International Studies, National University of Defense Technology, Nanjing, China; ^2^Institute of Education, Nanjing University, Nanjing, China

**Keywords:** critical cross-cultural literacy, individual identity, repertoire, systemic functional linguistics, cultural globalization

## 1 Introduction

The recent paper entitled *Repertoire construction for critical cross-cultural literacy of English majors: based on the research paradigm of systemic functional linguistics* by Zhao and Lu (2022) provides insightful pedagogical strategies for applying systemic functional linguistics (SFL) in English language teaching, which is a trend (Kramsch and Zhu, 2016; Schwartz et al., 2021) that has recently gained worldwide attention. The article aims to enhance English learners' (Els) critical cross-cultural literacy (CCCL), a new definition that emphasizes critical abilities and awareness in the cross-cultural education. To demonstrate the profound interplay between identity construction and linguistic coding, the authors employ the concept of individuation. They validate Ting-Toomey's (2020) classification of eight identities from the identity negotiation perspective (INP), and prove that Marxist dialectical philosophy and SFL's language viewpoint (Halliday et al., 2015; Hu, 2016) are feasible when studying and analyzing classroom discourse. The process involves a dual perspective, “recognizing” textbook authors' discourse strategy and “realizing” the coding orientation according to their identities to construct individual CCCL repertoires (Bernstein, 1996). We believe the theoretical, methodological, and practical contributions of this study make it an invaluable resource for English language educators and scholars alike.

## 2 Theoretical contribution

Credits should be given to the authors for their theoretical contribution in the combination of linguistic and pedagogical fields. Specifically, their insight in INP proves effective to be integrated into and refine the SFL appraisal system adopted as the theoretical framework in the article. In other words, the strategies of Els for utilizing semiotic resources in the SFL paradigm (Martin, 2009) are enriched to recognize the identities implied in the texts and realize repertoire construction for CCCL. As many scholars have noted, foreign language learners are “persons-in-context” (Ushioda, 2009), constantly interacting with situational affordances. To echo this aspect in this research, primary identities (culture, ethnic, gender, and personal identities) and situational identities (role, relational, facework, and symbolic interaction identities) (Ting-Toomey, 2020) are used to make a clearer depiction between the two-way process of individuation and affiliation. This approach facilitates Els' dialectical analysis of textual discourse, deconstructing the inter-subject relationship (Lu, 2011), and selecting linguistic codes based on their cultural experiences at the specific lexical and grammatic layer, thereby achieving the purpose of “alliance” in a two-way activity of “input” and “expression” (Saville-Troike, 2019).

## 3 Methodological innovation

This paper's noteworthy contribution by presenting a novel appraisal system that can serve as a valuable methodological tool for scholars. Drawing on the concept that Language systems are organized hierarchically by strata, rank, and metafunction, referred to as realization (Martin, 2009; Gebhard et al., 2013, 2014), the authors align three subsystems of this appraisal system—namely attitude, graduation, and engagement—with their corresponding components, including attitude type, attitude strength, and viewpoint source of discourse evaluation, respectively (Martin and White, 2005). Notably, this argument exhibits a cohesive unity between theory and text-based indicators, significantly enhancing the appraisal system's ability to identify strengths and weaknesses in classroom discourse strategies through codes. This systematic SFL approach in instructional settings offers valuable insights for researchers seeking to evaluate similar phenomena. Overall, we highly recommend this paper for its rigorous and thoughtful analysis, which will undoubtedly be of benefit to both scholars and practitioners in the fields of linguistics and language education.

## 4 Practical meaning

The ongoing evolution of language and culture highlights the importance of adopting a holistic approach to teaching linguistic skills, one that avoids the pitfalls of myopic focus on grammar and vocabulary, which may lead to the formation of “cultural stereotypes” amongst learners in higher education with unbalanced knowledge reserves and application abilities in China (Gao and Zhou, 2008). Such an approach risks impeding the development of students' CCCL, which is necessary for navigating and comprehending the constantly evolving language and culture of a given community. Therefore, cultivating students' CCCL is essential for equipping them with the communication skills required to engage meaningfully with the complex and perpetually evolving landscape of language and culture.

The practical meaning of this study is significant for the development of more sophisticated pedagogical models and instructional activities aimed at enhancing students' CCCL. Furthermore, the findings have important practical applications for educational administrators responsible for selecting appropriate teaching materials and formulating teaching guidance. Drawing on a macro-level SFL approach, this research emphasizes an objective perspective on language reality (Scollon and Scollon, 2001; Kramsch and Zhu, 2016), acknowledges individual developmental trajectories of language acquisition, and highlights the applicability of social discourse. This methodology aligns well with China's growing emphasis on cross-cultural education and provides guidance for integrating SFL into specific language teaching strategies such as writing, listening, speaking, and reading (Oliveira and Smith, 2019). Furthermore, Els can leverage the appraisal system to critically perceive underlying identities and orient codes toward them. We contend that the linguistic approach to pedagogy proposed by the authors is not only applicable to China but also has far-reaching impact beyond its borders.

## 5 Discussion

Despite the aforementioned merits, this study's validity requires further verification. Firstly, the generalization of the findings requires meticulous examination. Although a questionnaire survey was executed, the sample size was relatively small and failed to differentiate among “double first-class” universities in China. Given the substantial disparities among these institutions and their respective emphases on English pedagogy, the representativeness of the selected samples warrants deeper deliberation and critical scrutiny. Secondly, it is reasonable to acknowledge the lack of consideration accorded to the trend toward multimodal teaching in the evaluation system. Multimodal discourse encompasses all modes involving communication, such as words, pictures, music, colors, and techniques. Teachers can harness these modes to activate students' diverse emotions and facilitate their acquisition of English (Faigley et al., 2002). Hence, the extension of the research results of this paper to encompass multimodal discourse analysis aligns more comprehensively with current teaching model developments and would conduce positively to this research domain.

In conclusion, the study conducted by Zhao and Lu (2022) offers significant insights pertaining to SFL approach to pedagogy. Building upon this foundation, we advocate for the undertaking of further research efforts in this field. Consequently, this paper serves as a promising precursor, with the potential to catalyze a more comprehensive and informative discussion in the emerging research domain of the integration of linguistics and pedagogy.

## Author contributions

HM and JS drafted the General Commentary. JH and JS did the reviewing and editing for the text. All authors contributed to the article and approved the submitted version.

## References

[B1] Bernstein B. (1996). Pedagogy, Symbolic Control and Identity: Theory, Research, Critique. London: Taylor and Francis.

[B2] Faigley L. Kress G.R. Leeuwen T.M. (2002). Multimodal discourse: the modes and media of contemporary communication. Coll. Comp. Commun. 54:318. 10.2307/1512155

[B3] Gao Y. H. Zhou Y. (2008). English learning and learners' identity development in junior college years: evidence from a longitudinal study in five universities. For. Lang. Educ. Res. 6, 38–47.

[B4] Gebhard M. Chen I. A. Britton L. (2014). “Miss, nominalization is a nominalization:” English language learners' use of SFL metalanguage and their literacy practices. Linguist. Educ. 26, 106–125. 10.1016/j.linged.2014.01.003

[B5] Gebhard M. Chen I. A. Graham H. Gunawan W. (2013). Teaching to mean, writing to mean: SFL, L2 literacy, and teacher education. J. Second Lang. Writ. 22, 107–124. 10.1016/j.jslw.2013.03.005

[B6] Halliday M. A. K. He Y. X. Yang B. J. (2015). Marxism orientation of systemic functional linguistics: an interview with M. A. K. Halliday. Contemp. For. Lang. Stud. 7, 1–4+79.

[B7] Hu Z. L. (2016). A return to their cradle: the Chinese roots of Halliday's theories. For, Lang. Res. 159, 9–13+112.

[B8] Kramsch C. Zhu H. (2016). “Language and culture in ELT,” in The Routledge Handbook of English Language Teaching, ed. G. Hall (New York, NY: Routledge), 38–50.

[B9] Lu D. Y. (2011). The individuation vector of the architecture of language: the new SFL approach to language heterogeneity. For. Lang. Res. 2, 14–19.

[B10] Martin J. R. (2009). “Realization, instantiation and individuation: some thoughts on identity in youth justice conferencing,” in Proceedings of 36th SFLC (Beijing).

[B11] Martin J. R. White P. R. R. (2005). The Language of Evaluation: Appraisal in English. New York, NY: Palgrave Macmillan.

[B12] Oliveira L. Smith B. (2019). “Systemic functional linguistics in teacher education,” in Oxford Research Encyclopedia of Education. Available online at: https://oxfordre.com/education/view/10.1093/acrefore/9780190264093.001.0001/acrefore-9780190264093-e-494 (accessed May 31, 2024).

[B13] Saville-Troike M. (2019). Introducing Second Language Acquisition. Beijing: Foreign Language Teaching and Research Press.

[B14] Schwartz K. D. Exner-Cortens D. McMorris C. A. Makarenko E. Arnold P. Van Bavel M.. (2021). COVID-19 and student well-being: stress and mental health during return-to-school. Can. J. Sch. Psychol. 36, 166–185. 10.1177/08295735211001653 34040284 PMC8114331

[B15] Scollon R. Scollon S. W. (2001). Intercultural Communication: A Discourse Approach, Second Edn. Oxford: Blackwell Publishers Inc.

[B16] Ting-Toomey S. (2020). Communicating Across Cultures. Shanghai: Shanghai Foreign Language Education Press.

[B17] Ushioda E. (2009). “A person-in-context relational view of emergent motivation, self and identity,” in Motivation, Language Identity, and the L2 Self, eds. Z. Dörnyei, and E. Ushioda (Bristol: Multilingual Matters), 215–228.

[B18] Zhao R. Lu D. (2022). Repertoire construction for critical cross-cultural literacy of English majors: based on the research paradigm of systemic functional linguistics. Front. Psychol. 13:906175. 10.3389/fpsyg.2022.906175 35832913 PMC9273006

